# The Writer's Reasons for Not Eating Animal Food

**Published:** 1811-10

**Authors:** 


### *The Writer's Reasons for not Eating Animal Food.*

(Mouth. Mag.)

1. BECAUSE being mortal himself, and holding his life on the same uncertain and precarious tenure as all other sensitive beings, he does not feel himself justified by any supposed superiority or inequality of condition, in destroying the vital enjoyment of any other mortal, except in the necessary defence of his own life.

II.—Because the desire of life is so paramount, and so affectingly cherished in all sensitive beings, that he cannot reconcile it to his feelings to destroy, or become a voluntary party in the destruction of any living creature, however much in his power, or apparently insignificant.

III.—Because he feels an utter and unconquerable repugnance against receiving into his stomach the flesh or juices of deceased animal organization.

IV.—Because he feels the same abhorrence against devouring flesh in general, that he hears carnivorous men express against eating human flesh, or the flesh of dogs, cats, horses, or other animals, which in some countries it is not customary for carnivorous men to devour.

V.—Because Nature appears to have made a superabundant provision for the nourishment of animals in the saccharine matter of roots and fruits; in the farinaceous matter of grain, seed, and pulse, and in the oleaginous matter of the stalks, leaves, and pericarps of numerous vegetables.

VI.—Because the destruction of the mechanical organization of vegetables inflicts no sensitive suffering, nor violates any moral feeling; while vegetables serve to sustain his health, strength, and spirits, above those of most carnivorous men.

VII.—Because during thirty years of rigid abstinence from the flesh and juices of deceased sensitive beings, he finds that he has not suffered a day's serious illness; that his animal strength and vigour have been equal, or superior to that of other men; and that his mind has been fully equal to numerous shocks, which it has had to encounter from malice, envy, and various acts of turpitude in his fellow-men*.

---

* The Author at twelve years old, when a school-boy at Chiswick, abstained from eating animal food from a cause which it is said led Dr. Franklin

VIII.—Because observing that carnivorous propensities among animals are accompanied by a total want of sympathetic feelings, and humane sentiments, as in the hyæna, the tyger, the vulture, the eagle, the crocodile, and the shark; he conceives that the practices of those carnivorous tyrants afford no worthy example for the imitation or justification of rational, reflecting, and conscientious beings.

IX.—Because he observes that carnivorous men, unrestrained by reflection or sentiment, even refine on the cruel practices of the most savage animals; and apply their resources of mind and art to prolong the miseries of the victims of their appetites, skinning, roasting, and boiling animals alive, and torturing them without reservation or remorse, if they thereby add to the variety or the delicacy of their carnivorous gluttony.

X.—Because the natural sentiments and sympathies of human beings, in regard to the killing of other animals, are generally so averse from the practice, that few men or women could devour the animals which they might be obliged themselves to kill: yet they forget, or affect to forget, the living endearments or dying sufferings of the creature, while they are wantoning over his remains.

XI.—Because the human stomach appears to be naturally so averse from receiving the remains of animals, that few could partake of them if they were not disguised and flavoured by culinary preparation; yet rational creatures ought to feel that the prepared substances are not the less what they truly are, and that no disguise of food, in itself loathsome, ought to delude the unsophisticated perceptions of a considerate mind.

XII.—Because the forty-seven millions of acres in England and Wales would maintain in abundance as many human inhabitants, if they lived wholly on grain, fruits, and vegetables; but they sustain only twelve millions scantily, while animal food is made the basis of human subsistence.

XIII.—Because animals do not present or contain the substance of food in mass, like vegetables; every part of their economy being subservient to their mere existence, and their

---

Franklin to resume the practice. He saw a fish opened which had small fish within it, recently devoured; and when that fish was afterwards brought to table, he was forcibly struck with the idea of eating the very animal, which but yesterday had been devouring others. The practice of the fish was, he felt, that of a creature without reason or humanity, and no justification to him for doing what he thought wrong. His appetite also revolted at the idea of eating part of a creature so lately and so palpably enjoying itself in its own element. He therefore excused himself, and has to this time persevered in rigid abstinence.

entire entire frames being solely composed of blood necessary for life, of bones for strength, of muscles for motion, and of nerves for sensation.

XIV.—Because the practice of killing and devouring animals can be justified by no moral plea, by no physical benefit, nor by any allegation of necessity, in countries where there is abundance of vegetable food; and where the arts of gardening and husbandry are favoured by social protection, and by the genial character of the soil and climate.

XV.—Because whenever the number and hostility of predatory land animals might so tend to prevent the cultivation of vegetable food, as to render it necessary to destroy, and perhaps, to eat them, there could in that case exist no necessity for destroying the animated existences of the distinct elements of air and water; and, as in most civilized countries, there exists no land animals besides those which are purposely bred for slaughter or luxury, of course the destruction of animals, birds, and fish, in such countries must be ascribed either to unthinking wantonness or carnivorous gluttony.

XVI.—Because the stomachs of loco-motive beings appear to have been provided for the purpose of conveying about with the moving animal, nutritive substances, analogous in effect to the soil in which are fixed the roots of plants, and consequently nothing ought to be introduced into the stomach for digestion and for absorption by the lacteals, or roots of the animal system, but the natural bases of simple nutrition, as the saccharine, the oleaginous, and the farinaceous matter of the vegetable kingdom.

COMMON SENSE.

*July 27th,* 1811.

